# What about the environment? Leveraging multi-omic datasets to characterize the environment’s role in human health

**Published:** 2021

**Authors:** Kristin Passero, Shefali Setia-Verma, Kimberly McAllister, Arjun Manrai, Chirag Patel, Molly Hall

**Affiliations:** Huck Institutes of the Life Sciences, The Pennsylvania State University, University Park, PA 16802; Department of Genetics and Institute for Biomedical Informatics, University of Pennsylvania, Philadelphia, PA 19104; Genes, Environment, and Health Branch, Division of Extramural Research and Training, National Institute of Environmental Health Sciences, P.O. Box 12233 (MD EC-21), Research Triangle Park, NC 27709; Computational Health Informatics Program, Boston Children’s Hospital, Department of Biomedical Informatics, Harvard Medical School, Boston, MA 02115; Department of Biomedical Informatics, Harvard Medical School, Boston, MA 02115; Department of Veterinary and Biomedical Sciences, The Pennsylvania State University, University Park, PA 16802

**Keywords:** Environment, Health Outcomes, Multi-omics

## Abstract

The environment plays an important role in mediating human health. In this session we consider research addressing ways to overcome the challenges associated with studying the multifaceted and ever-changing environment. Environmental health research has a need for technological and methodological advances which will further our knowledge of how exposures precipitate complex phenotypes and exacerbate disease.

## 1. The complexities of environmental health research

The environment is increasingly seen as a casual or moderating factor that governs aspects of complex disease etiology (Hall, Moore, & Ritchie, 2016; Manrai et al., 2017). Since there is a great breadth of environmental risk factors, researchers classify exposures into three categories: (Stingone, Buck Louis, et al., 2017; Wild, 2012) internal exposures arising from endogenous processes (e.g. metabolism, inflammation), intrinsic qualities (e.g. body morphology), or microorganisms living in or on an individual (e.g. microbes colonizing the gut) that affect the body’s cellular environment (Wild, 2012). Specific external exposures are extrinsic and “target” the body directly. Examples include infectious agents, diet and substance use, pollutants, and occupational exposures (Martin Sanchez, Gray, Bellazzi, & Lopez-Campos, 2014; Wild, 2012). Lastly, general external exposures are broad characteristics, such as which geography and climate a person resides in, socioeconomic indicators, or psychosocial exposures, that affect both the individual and, to a degree, the experience of internal and specific external exposures (Wild, 2012). Household income, work-life balance, healthcare access, or home rurality are general external exposures.

A comprehensive assessment of environmental risk factors remains challenging as the environment is dynamic. Exposure presence and intensity change over time. Environmental risk is a cumulative measure acquired throughout the lifespan and beginning from conception (Manrai et al., 2017; Stingone, Buck Louis, et al., 2017). Longitudinal investigation of exposures is crucial for research investigating vulnerability periods, such as the prenatal period, where exposures impart their most salient effects on health. The within-person heterogeneity of exposures is a major limitation in the field of human exposure research, as timing and intensity may be difficult to capture without consistent monitoring (Manrai et al., 2017; van Tongeren & Cherrie, 2012). Sources of environmental data are diverse. Environmental data may be obtained from surveys or can rely on a collection of ‘omics level data, such as the metabolome and the microbiome, when quantifying measures such as exogenous chemical exposure, internal metabolism, or gut microbial diversity. Other sources of information about environmental circumstances may come from purchasing history, food expenditures, mobile phones, social media, or home sensors (Martin Sanchez et al., 2014; van Tongeren & Cherrie, 2012).

Another limitation in environmental health research is the relative dearth of data analytic tools, databases and ontologies, and standardized practices which would aid in the assessment of high-dimensional exposure data (Bocato, Bianchi Ximenez, Hoffmann, & Barbosa, 2019; Manrai et al., 2017; Martin Sanchez et al., 2014; Stingone, Buck Louis, et al., 2017). Researchers seeking to utilize big environmental data would benefit from the development of methods and infrastructure to investigate environmental underpinnings of disease. This includes the curation of high-information environmental datasets (e.g. the HELIX study (Vrijheid et al., 2014)), analytical techniques to assess multivariate, longitudinal data or environmental mixtures (Manrai et al., 2017; Patel, 2017), and curation of database/development of ontologies for known environmental risk factors and their associations (Manrai et al., 2017; Martin Sanchez et al., 2014).

## 2. Progress made in environmental health research

Environmental health research is a multidisciplinary field and its past successes have utilized various approaches and data types. A study of gene-by-environment interaction found that subjects sharing regional ancestry but living in different regions, showed many differentially expressed genes, whose expression was correlated with fine-scale air pollution (Favé et al., 2018). In a closer look, they identified four quantitative trait loci where transcription was moderated by pollution level (Favé et al., 2018). Other approaches have leveraged environment-wide datasets and found associations between exposures and phenotypes. For example, an environment-wide association study (EWAS) found that blood serum antioxidants, vitamin D, and intense physical activity were associated with abdominal obesity in both sexes (Wulaningsih et al., 2017), and a meta-analysis of EWAS performed on the National Health and Nutrition Examination Surveys from 1999–2012 identified alcohol consumption and urinary cesium as associated with systolic and diastolic blood pressure respectively (McGinnis, Brownstein, & Patel, 2016). The microbiome is increasingly seen as a player in human health (Young, 2017). An investigation of Type I Diabetes onset in infants found that prior to diagnosis, gut microbial diversity decreased and microbe metabolite production reflected a shift towards nutrient transport rather than biosynthesis (Kostic et al., 2015). Machine learning (ML) methods have been applied to probe how pollutant exposures within urban areas affect academic performance (Stingone, Pandey, Claudio, & Pandey, 2017). Another study used ML to create environmental risk scores for oxidative stress which were associated with cardiovascular phenotypes (Park, Zhao, & Mukherjee, 2017).

Metabolomics is useful when assessing environmental risk factors as it can detect both internal exposures (e.g. proinflammatory molecules) and chemicals or toxins (Bloszies & Fiehn, 2018). Computational tools to enable untargeted metabolomics studies, which will aid researchers seeking to agnostically profile the environment, are emerging (Domingo-Almenara et al., 2019; Pirhaji et al., 2016). Other open-source software developed for the quality-control, analysis, and visualization of general environment-wide data (Hernandez-Ferrer et al., 2019; Lucas et al., 2019) are also becoming available to researchers. Future projects will benefit from the curation of environment-wide databases for blood (Barupal & Fiehn, 2019), urine (Jia et al., 2019), and the indoor built environment (Dong et al., 2019) as guides for future, larger-scale metabolomics projects. Finally, the most comprehensive assessment of environment may be achieved through rigorous biomonitoring. Jiang and colleagues (2018) conducted an impressive study by fitting participants with wearable devices which collected longitudinal data on climate, biotic, and abiotic factors. They found the human environment of microbial and chemical exposure varied widely across geographical location and season, even within the same individual (Jiang et al., 2018).

There is much evidence that the environment impacts human health, with disease risk arising from many sources: pollutants, industrial chemicals, lifestyle habits, social climate, etc. Yet the challenges of collecting and analyzing environmental data remain. Different sources of environmental data may need different methodological standards and techniques for effective research. Thus, researchers need user-friendly tools to handle pre-processing, quality assessments, and analysis of various data types. There also remain the questions of which environmental data are most informative when predicting health outcomes, and how we can integrate these various sources of data to define environment-wide risk. There are many opportunities for researchers to develop or improve existing methodologies and advance environmental health research.

## 3. In this session

Demonstrating the breadth found within environmental health research, our selected publications address key areas of environmental health research: (1) metabolomic profiling and pipeline development and (2) the role of sociodemographic in the prediction of complex health outcomes.

Aguilar, McGuigan, and Hall have developed a semi-automated pipeline for processing and analyzing NMR data. Their method uses open-source software, making it accessible to researchers and easy to document, thereby improving reproducibility and replication capabilities. After applying their pipeline to assess how smoking perturbs human metabolism, they identified associations between various metabolites which past research suggests are implicated in cardiac, pulmonary, and neural diseases. Furthermore, metabolites showing ostensibly differential concentrations between smokers and non-smokers were used as input for a random forest model. This technique found metabolic heterogeneity between and within smoking classes, identifying several unique metabolic profiles which distinguished subsets of smokers and non-smokers. Their study emphasizes how a single exposure, such as smoking, may precipitate complex phenotypic outcomes. Furthermore, it leveraged the metabolome in a joint assessment of the internal and external environment. Smoking was linked to changes in the internal environment, which may in turn affect physiology. Additionally, profiling the metabolome identified within smokers an exogenous pollutant absorbed by tobacco plants. Aguilar et al. highlight how multiple sources of environmental risk may act in concert to develop complex phenotypes.

While the former study evaluates how an acute environmental risk factor is associated with multiple metabolic phenotypes, the environment also exerts influence at a societal and geographical level. Makridis, Strebel, and Alerovitz assessed how different geographic granularities of sociodemographic data affect prediction of mortality in veterans hospitalized due to COVID-19. Their social variables included ZIP-code-level, county-level, or state-level population density, healthcare access, and distributions of age, race/ethnicity, occupation, and education. They noted that in linear models using comparable demographic variables measured county-level or state-level, demographics differed in the effect sizes and significance in association with COVID-19 cumulative cases and deaths. When predicting veteran mortality attributed to COVID-19 using a linear XGBoost algorithm, county-level and ZIP-code level data had negligible differences in prediction accuracy, yet outperformed state-level prediction. Yet interestingly, the features most important in the county-level model differed from that of the ZIP code-level model. The granularity of the environmental data is important when predicting outcomes in a region. Social environmental data may be collected at multiple hierarchies – e.g. state, county, ZIP code – and the demographics at each level may carry different information pertaining to health outcomes, which may be important when trying to design and implement public health policies.

Together, these papers highlight the nuanced relationship the environment has with human disease. The environment has an unavoidable influence on life yet remains difficult to characterize and quantify. It has many dimensions (e.g. internal, specific external, general external), a hierarchical organization (e.g. environment at the individual, home, neighborhood, county, etc. levels), and is dynamic which makes parsing the relevant components which contribute to disease risk challenging. Answering *what*, *when*, and *how* environmental factors affect health requires collecting data that reflects environmental diversity. This may be achieved by collecting environment-wide data covering multiple domains, capturing exposures longitudinally, or, as Makridis et al. imply, considering environmental data at different organizational hierarchies. Simultaneously, researchers must develop and evaluate ways to handle data heterogeneity, model environmental mixtures and interactions, and assess risk at various levels.
